# Functional Analysis of Brain Imaging Suggests Changes in the Availability of mGluR5 and Altered Connectivity in the Cerebral Cortex of Long-Term Abstaining Males with Alcohol Dependence: A Preliminary Study

**DOI:** 10.3390/life11060506

**Published:** 2021-05-30

**Authors:** Yo-Han Joo, Jeong-Hee Kim, Hang-Keun Kim, Young-Don Son, Paul Cumming, Jong-Hoon Kim

**Affiliations:** 1 Neuroscience Research Institute, Gachon University, Incheon 21565, Korea; yhjoo@gachon.ac.kr (Y.-H.J.); jhkim1104@bme.gachon.ac.kr (J.-H.K.); dsaint@gachon.ac.kr (H.-K.K.); 2 Department of Biomedical Engineering, College of Health Science, Gachon University, Incheon 21936, Korea; 3 Gachon Advanced Institute for Health Science and Technology, Graduate School, Gachon University, Incheon 21565, Korea; 4 Department of Nuclear Medicine, Inselspital, University of Bern, CH-3010 Bern, Switzerland; paul.cumming@insel.ch; 5 School of Psychology and Counselling, Queensland University of Technology, Brisbane, QLD 4059, Australia; 6 Gil Medical Center, Department of Psychiatry, Gachon University College of Medicine, Gachon University, Incheon 21565, Korea

**Keywords:** metabotropic glutamate receptor-5, positron emission tomography, [^11^C]ABP688, functional magnetic resonance imaging, alcohol dependence

## Abstract

Direct in vivo evidence of altered metabotropic glutamate receptor-5 (mGluR5) availability in alcohol-related disorders is lacking. We performed [^11^C]ABP688 positron emission tomography (PET) and resting-state functional magnetic resonance imaging (rs-fMRI) in prolonged abstinent subjects with alcohol dependence to examine alterations of mGluR5 availability, and to investigate their functional significance relating to neural systems-level changes. Twelve prolonged abstinent male subjects with alcohol dependence (median abstinence duration: six months) and ten healthy male controls underwent [^11^C]ABP688 PET imaging and 3-Tesla MRI. For mGluR5 availability, binding potential (BP_ND_) was calculated using the simplified reference tissue model with cerebellar gray matter as the reference region. The initial region-of-interest (ROI)-based analysis yielded no significant group differences in mGluR5 availability. The voxel-based analysis revealed significantly lower [^11^C]ABP688 BP_ND_ in the middle temporal and inferior parietal cortices, and higher BP_ND_ in the superior temporal cortex in the alcohol dependence group compared with controls. Functional connectivity analysis of the rs-fMRI data employed seed regions identified from the quantitative [^11^C]ABP688 PET analysis, which revealed significantly altered functional connectivity from the inferior parietal cortex seed to the occipital pole and dorsal visual cortex in the alcohol dependence group compared with the control group. To our knowledge, this is the first report on the combined analysis of mGluR5 PET imaging and rs-fMRI in subjects with alcohol dependence. These preliminary results suggest the possibility of region-specific alterations of mGluR5 availability in vivo and related functional connectivity perturbations in prolonged abstinent subjects.

## 1. Introduction

Recent years have seen the emergence of a greater understanding of the contribution of glutamatergic neurotransmission to the pathophysiology of the symptoms and signs related to alcohol dependence [1,2,3], a chronic disorder characterized by compulsive alcohol-seeking behaviors and progressive psychosocial dysfunction. The development of alcohol dependence occurs through multiple stages such as reward seeking/binge drinking, withdrawal, and cue-induced craving [4]. Glutamatergic signaling is mainly involved in incentive salience/binge drinking and alcohol craving [5]. Indeed, microdialysis analysis showed that alcohol modulates the release of glutamate by parvalbumin-expressing interneurons in the rat medial prefrontal cortex (mPFC) [6]. Other rat studies showed that the glutamatergic signaling in the infralimbic subregion of the PFC facilitated extinction of alcohol-seeking behaviors [7], while activity-dependent ablation of neurons in parts of the PFC induced excessive alcohol seeking in rats [8]. In addition, pharmacological blockade of glutamate receptors in the basolateral amygdala and nucleus accumbens reduced cue-induced reinstatement of alcohol in rats [9]. Overall, a range of preclinical studies emphasize the role of glutamatergic signaling in the prelimbic and infralimbic subregions of the PFC and subcortical limbic circuits in the components of incentive salience and cue-induced reinstatement of alcohol. In particular, the metabotropic glutamate receptors (mGluRs), which mediate synaptic neurotransmission through G-protein-coupled intracellular signaling pathways, are receiving increasing attention in this context, since pharmacological manipulation of mGluRs results in profound effects on alcohol-seeking behaviors in animal models [10,11]. The mGluRs are also implicated in the altered neuronal plasticity in brain regions critical for addiction behaviors [12,13]. Consequently, mGluRs are being actively explored as useful markers for alcohol dependence, and as therapeutic targets for treatment of alcohol-related disorders [11].

Among the several mGluR subtypes, the type I metabotropic glutamate receptor-5 (mGluR5), which is predominantly expressed in the cortex, limbic regions, and the basal ganglia, is of significant interest in relation to alcohol dependence [10]. Previous rodent studies have shown that mGluR5 antagonists successfully attenuate cue-conditioned alcohol-seeking behavior, as well as the rewarding effects of alcohol measured in a self-administration paradigm [14,15]. In addition, genetic deletion of mGluR5 in mice resulted in reduced alcohol consumption and increased behavioral sensitivity to the acute central effects of alcohol [16]. Furthermore, allelic variants of mGluR5 have been linked with the propensity for alcohol dependence in humans [17]. These findings all point to a critical involvement of mGluR5-mediated neurotransmission in the pathophysiology of alcohol dependence.

Previous positron emission tomography (PET) studies with [^11^C]ABP688 have shown that mGluR5 availability is significantly lower in nicotine dependence [18,19] and cocaine addiction [20], suggesting that long-term use of these drugs is associated with a decrease in mGluR5 availability. There are inconsistent findings of altered mGluR5 expression in brain of humans with alcohol dependence. One postmortem autoradiographic study reported 30–40% higher [^3^H]quisqualic acid binding to mGluR5 in the CA2 layer of the hippocampus in alcohol-dependent subjects compared with controls [21]. In one of the few in vivo PET imaging studies aiming to quantify mGluR5 availability in subjects with alcohol dependence, Akkus et al. [22] reported significantly higher mGluR5 availability in temporal lobe regions in male patients with alcohol use disorder, who had been abstinent for on average 25 (standard deviation (SD) = 18) days. On the other hand, significantly lower mGluR5 availability in corticolimbic areas (frontal, parietal, temporal, and cingulate cortices, insula, thalamus, and caudate) was observed in alcohol-dependent subjects examined using [^18^F]FPEB PET within two weeks of supervised abstinence [23]. A recent publication reported that alcohol-dependent subjects showed sustained recovery of mGluR5 availability in corticolimbic regions during a six-month period of alcohol abstinence [24].

The goal of treatment for alcohol dependence is to achieve longer-term abstinence and psychosocial recovery [25]. Therefore, it is crucial in PET studies to examine subjects with an extended period of abstinence to elucidate the underlying adaptive neuronal mechanisms and functional brain changes that may be associated with enhanced self-control and motivation, to help patients in overcoming their urge to use alcohol and to facilitate extinction learning [26]. Moreover, functional changes at the neuroreceptor level, which can be measured using PET molecular imaging techniques, are ultimately related to systems-level changes in neural circuitry, representing specific behavioral phenotypes [27,28]. However, the relationship between subtle alterations in mGluR5 availability and neural systems-level perturbations is unknown in alcohol dependence. Therefore, combining molecular PET imaging with a specific mGluR5 ligand and functional magnetic resonance imaging (fMRI) measurement of resting-state functional connectivity could provide insight into the relationship between mGluR5-mediated neurotransmission and modulation of neural systems-level changes underlying alcohol dependence.

As such, the purpose of the present study was to quantify in vivo mGluR5 availability using PET with the selective allosteric radioligand [^11^C]ABP688 in male subjects abstinent from alcohol for an extended period of time and in matched controls. Our goal was to search for alterations in mGluR5 availability in these prolonged abstinent subjects compared to controls. To examine the functional significance of alterations in mGluR5 availability, we also performed resting-state fMRI (rs-fMRI) in the same subjects and conducted seed-based functional connectivity analysis using rs-fMRI data with regions derived from quantitative PET imaging analysis as the seeds.

## 2. Materials and Methods

### 2.1. Participants

The study protocol was approved by the Institutional Review Board of the Gachon University Gil Medical Center, and written informed consent was obtained from all participants after a full explanation of the study procedure. All procedures complied with the ethical standards of the relevant national and institutional committees on human experimentation and with the Helsinki Declaration of 1975, as revised in 2008. The enrollment criteria for subjects with alcohol dependence were as follows: (i) male subjects aged 30–55 years; (ii) Diagnostic and Statistical Manual of Mental Disorders, Fourth Edition (DSM-IV) lifetime criteria for alcohol dependence [29] and no other current Axis I diagnosis; (iii) abstinence of alcohol for at least four weeks; (iv) no past or current dependence on other substances (nicotine use was allowed); (v) no concurrent medical or neurological disorders to affect the central nervous system; (vi) no current use of medications known to affect the central nervous system (except for nicotine use). The psychiatric diagnosis was established using the Structured Clinical Interview for DSM-IV [30]. Twelve male subjects were enrolled and completed the study (Table 1). They had a mean age of 45.7 (SD = 7.2) years and a mean duration of illness of 7.2 (SD = 3.9) years, defined as the period from the first diagnosis of alcohol dependence. Prior to abstinence, the subjects reported consuming a mean of 13.5 (SD = 6.8) standard drinks per single drinking session. The reported drinking frequency per week was 3.9 (SD = 1.5). A standard drink amounts to 14 g of pure alcohol [31]. The calculations were based on this international standard and traditional Korean drinks (a bottle of Korean liquor (soju: about 90 mL) with 19% alcohol, a bottle of Korean rice wine (makgeolli: about 750 mL) with 6% alcohol, and a bottle of Korean beer (about 350 mL) with 4.5% alcohol) [31]. The mean duration of alcohol abstinence was 28.4 (SD = 43.0) months (median: six months; range: 1.5 to 121.5 months). Nine subjects were smokers, and three were nonsmokers, of whom one was an ex-smoker with 16 months of abstinence. The mean number of cigarettes smoked per day was 10.8 (SD = 9.5). None of the subjects were receiving benzodiazepines at the time of enrollment. However, 11 subjects had received benzodiazepines for detoxification treatment during early abstinence. None of the subjects were taking anticraving medications such as acamprosate or naltrexone at the time of enrollment. All subjects were recruited from local psychosocial rehabilitation centers, and were being monitored regularly for relapse based on urine and blood tests in the affiliated hospitals. A breathalyzer test was administered to confirm a breath alcohol concentration (BrAC) of zero at the time of enrollment.

To compare neuroimaging findings between abstinent subjects with alcohol dependence and healthy subjects, we recruited ten age-matched healthy male control subjects, who met the criteria of no current or past psychiatric, neurological, or medical disorders, and with no current use of medications known to affect the central nervous system (except for nicotine use). These subjects provided written informed consent, and underwent the same PET and rs-fMRI protocols as the subjects of the alcohol group. The mean age of the control subjects was 39.9 (SD = 7.9) years. None of the control subjects were known former (recovered) alcoholics; seven were nondrinkers and three were social drinkers reporting on average 0.94 (SD = 0.07) standard drinks per week. Five subjects were smokers, and five were nonsmokers, of whom one was an ex-smoker with 13 years of abstinence. None of the participants showed any gross structural abnormalities on brain MRI, which was confirmed by a board-certified radiologist. The control subjects were recruited through local advertisements.

### 2.2. Clinical Assessments

The severity of alcohol dependence was assessed using the Michigan Alcoholism Screening Test (MAST) [32]. Alcohol craving was assessed using the Obsessive Compulsive Drinking Scale (OCDS) [33]. The MAST and OCDS were both standardized for the Korean population [34,35]. A total score was obtained for each scale.

### 2.3. Scan Protocol for [^11^C]ABP688 PET Imaging

Subjects were scanned with [^11^C]ABP688 using a Biograph 6 PET scanner (Siemens Medical Imaging Systems, Knoxville, USA). The tracer [^11^C]ABP688 was synthesized as previously described [36]. After a bolus injection of 607 (SD = 58) MBq [^11^C]ABP688 with a molar activity of 20.2 (SD = 9.1) GBq/μmol, a dynamic list-mode emission recording was obtained lasting 60 min. A computed tomography (CT)-based transmission scan was performed just prior to the emission scan, and the calculated CT transmission map was used for PET attenuation correction. The [^11^C]ABP688 PET images were reconstructed using the two-dimensional ordered-subset expectation maximization (OSEM-2D) algorithm. The reconstructed PET images had a matrix size of 256 × 256 × 109 and a voxel size of 1.33 × 1.33 × 1.50 mm^3^. To calculate the [^11^C]ABP688 binding potential with respect to nondisplaceable compartment (BP_ND_), the emission data of [^11^C]ABP688 PET were reconstructed as 21 frames of increasing duration as follows: 2 × 15 s, 3 × 30 s, 3 × 60 s, 2 × 90 s, 3 × 120 s, 2 × 180 s, 4 × 300 s, and 2 × 600 s (total 60 min). BP_ND_, binding potential with respect to nondisplaceable compartment, refers to the ratio of specifically bound radioligand to that of nondisplaceable radioligand in living brain tissue at equilibrium, which is proportional to the ratio B_max_/K_D_ measured in vitro [37]. Attenuation, scatter, and decay time correction were applied for each frame.

### 2.4. Scan Protocol for Resting-State fMRI

Three-Tesla MRI (Magnetom Verio; Siemens, Erlangen, Germany) scans were performed using a three-dimensional T1-weighted magnetization-prepared rapid gradient echo (3D T1MPRAGE) sequence for structural brain imaging. The 3D T1MPRAGE images were acquired with the following parameters: repetition time = 1900 ms, echo time = 3.3 ms, inversion time = 900 ms, flip angle = 9°, voxel size = 0.5 × 0.5 × 1.0 mm^3^, matrix size = 416 × 512, and number of slices = 160.

For rs-fMRI recordings optimized for detecting changes in blood oxygen level-dependent (BOLD) signal, 3-Tesla rs-fMRI images were acquired in transaxial directions using echo planar imaging (EPI) with the following parameters: repetition time = 3000 ms, echo time = 30 ms, flip angle = 90°, pixel size = 3.5 × 3.5 mm^2^, thickness = 3.5 mm, matrix size = 72 × 72, and number of slices = 45. Twelve channels of a transmit-and-receive radiofrequency phase array head coil (iPAT, Siemens, Erlangen, Germany) were used. A total of 180 volumes were acquired for each subject’s session, which lasted nine minutes. The subjects were instructed to relax, lie still, and stay awake in the scanner. To minimize head movement, the head was comfortably restricted by sponges and subjects were instructed not to move their head during scanning.

### 2.5. Image Analysis

#### 2.5.1. [^11^C]ABP688 PET Imaging

**Figure 1:**
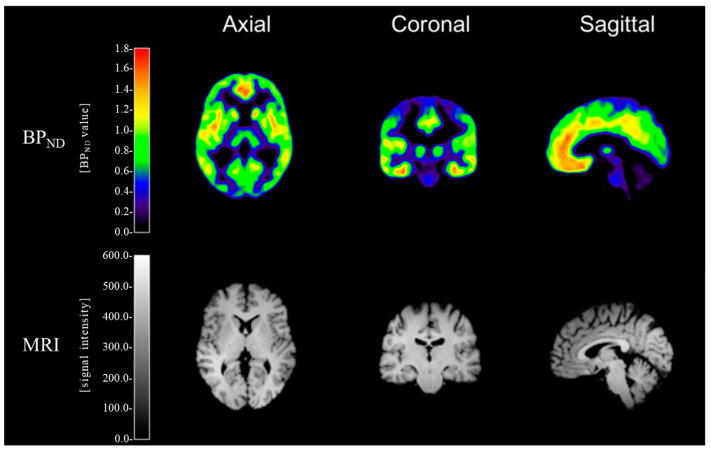
Representative examples of [^11^C]ABP688 BP_ND_ and 3-Tesla MR images of a control subject. (MNI coordinates: x = 3.0 mm, y = -19.5 mm, and z = 4.5 mm).

The 3D T1MPRAGE MRI images of each subject were coregistered to his PET images using statistical parameter mapping 8 (SPM8; Wellcome Trust Center for Neuroimaging, London, UK). The spatial normalization of the coregistered MRI images of each subject was performed on the Montreal Neurological Institute (MNI) template using SPM8, and the calculated transform was applied to the corresponding PET images. Time–activity curves of [^11^C]ABP688 PET were generated from dynamic PET images by frame-wise averaging of all voxels within each region of interest (ROI), which were coregistered to the corresponding MRI images. For the estimation of mGluR5 availability, [^11^C]ABP688 BP_ND_ was derived from each ROI using the simplified reference tissue model 2 (SRTM2) [38] with cerebellar gray matter as the reference region [39,40,41], based on the parameter estimation implemented in the PMOD software v3.2 (PMOD Technologies Ltd., Zürich, Switzerland). The cerebellar gray matter has been validated as a suitable reference region for [^11^C]ABP688 PET [42,43]. In addition, postmortem studies of mGluR5 mRNA or protein expression have reported that specific mGluR5 binding in cerebellar gray matter is negligible [44,45,46]. Moreover, in our study, there was no significant difference in cerebellar standard uptake value (SUV) between the alcohol and control groups (*p* = 0.45) (Supplementary Figure S1), nor was there any significant group difference in cerebellar gray matter volume (absolute volume: *p* = 0.92; relative volume: *p* = 0.76) (Supplementary Figure S1). Representative examples of [^11^C]ABP688 BP_ND_ and 3-Tesla MR images are shown in Figure 1. The BP_ND_ values were obtained in the 14 predefined ROIs using the automated anatomical labeling (AAL) program [47] and in the one predefined ROI using the manual mask. The manually defined mask of the ventral striatum, which is not defined on the AAL program, was drawn on the spatially normalized mean T1 MR image following previously defined divisions [48,49]. The ROIs were the anterior cingulate gyrus, superior frontal cortex, middle frontal cortex, inferior frontal cortex, superior parietal cortex, inferior parietal cortex, superior temporal cortex, middle temporal cortex, hippocampus, amygdala, thalamus, caudate, putamen, globus pallidus, and ventral striatum. These 15 ROIs were selected based on previous mGluR5 PET studies on alcohol dependence [19,23,24], and informed by literature implicating the regions as major nodes in a cortico-basal ganglia-limbic network in alcohol dependence [50].

#### 2.5.2. [^11^C]ABP688 BP_ND_ Seed-Based Resting-State fMRI

Preprocessing of the MRI images was performed using SPM8. The 3D T1MPRAGE image of each subject was segmented into gray matter, white matter, and cerebrospinal fluid (CSF) images and was coregistered to the rs-fMRI image. The rs-fMRI images were aligned to the first image, and the first five frames of the sequence were removed to eliminate the initial signal drift. The segmented T1 images were spatially normalized to the MNI template, and the same transform was applied to the corresponding rs-fMRI images. Both images were resampled to 3 mm isotropic voxels and smoothed by 3D Gaussian low-pass filter with 6 mm full width at half maximum (FWHM).

Denoising of the fMRI data was performed using linear regression to remove unwanted physiological and motion signals. The denoising steps were based on a default scheme implemented in the functional connectivity toolbox (CONN) software package (CONN v17.e; MIT McGovern Institute for Brain Research, Cambridge, MA, USA) (http://web.mit.edu/swg/software.htm (accessed on 30 March 2021)) [51]. The five principal components from white matter and CSF time series were extracted using the CompCor method [52] and then added as confounds in the denoising step. Realignment parameters in six degrees of freedom were also included as regressors to correct for motion. The bandpass filtering (0.008–0.09 Hz) and linear detrending were applied in this step.

To investigate the effect of altered mGluR5 availability on functional connectivity in subjects with alcohol dependence, we performed seed-to-ROI analysis of the rs-fMRI data between groups using the CONN software package. In the seed-to-ROI rs-fMRI analysis, seed regions were based on the peak voxel coordinates, i.e., clusters set with a statistical threshold, and they were derived from the voxel-based between-group comparisons of [^11^C]ABP688 BP_ND_ (BP_ND_-based seeds). The level of statistical threshold in this voxel-based analysis was defined as uncorrected *p* < 0.001 with a cluster size threshold of 20 voxels. For each subject, bivariate correlations between the seeds and ROIs were used for the first-level analysis of functional connectivity. A library of 106 predefined cortical and subcortical regions (FMRIB Software Library (FSL) Harvard–Oxford Atlas) [53] and 30 functional network regions (CONN’s Independent Component Analyses of the Human Connectome Project (HCP) dataset) [54] were used as the target ROIs in the seed-to-ROI rs-fMRI analysis.

### 2.6. Statistical Analysis

#### 2.6.1. [^11^C]ABP688 PET Imaging

The mean [^11^C]ABP688 BP_ND_ values were compared between the groups using ROI-based and voxel-based approaches. In the ROI-based analysis, right and left regions were summed, and mean BP_ND_ values (sum of BP_ND_ values/total voxel number of bilateral regions) were calculated as previously proposed [55,56], since there was no indication of BP_ND_ lateralization in our subjects (*t* = −0.93 to 1.30, *p* > 0.1). In the ROI-based analysis, between-group comparisons were performed using two-tailed t-tests. Effect sizes (Cohen’s *d*) were also calculated.

To assess the relationship between mGluR5 availability and clinical variables, we first examined the correlations between demographic variables (age and the number of cigarettes smoked per day) and [^11^C]ABP688 BP_ND_ values using ROI-based and voxel-based analyses. Then, the relationships between [^11^C]ABP688 BP_ND_ values and alcohol dependence-related variables were analyzed using ROI-based and voxel-based approaches with relevant variables as covariates, as appropriate. In the ROI-based analysis, relationships were analyzed using Pearson’s correlation coefficients. In the voxel-based analysis, voxel-wise linear regression analysis was conducted using spatially normalized [^11^C]ABP688 BP_ND_ images with clinical variables (duration of illness, abstinence period, MAST scores, and OCDS scores) as the regressors. A two-tailed *p* < 0.05 was considered statistically significant in the ROI-based analyses. The voxel-based analysis was performed using SPM8, and the level of statistical significance was defined as uncorrected *p* < 0.001 with a cluster size threshold of 20 voxels, as deemed acceptable and appropriate in previous PET imaging studies [57,58,59].

#### 2.6.2. Functional Connectivity Using Resting-State fMRI

The [^11^C]ABP688 BP_ND_ seed-based rs-functional connectivity was analyzed as follows. The correlations between the average time courses of the BP_ND_-based seeds to ROIs were calculated, and Fisher’s z-transformation was performed on the Pearson’s bivariate correlation coefficients. A two-sample t-test between the alcohol and control groups was then performed on the resultant correlation coefficients to examine group differences in seed-based rs-functional connectivity. The significance threshold was set at uncorrected *p* < 0.001.

## 3. Results

The clinical characteristics of the participants are presented in Table 1. Demographic characteristics and scan parameters did not differ between the alcohol and control groups. Age, smoking status, and the number of cigarettes smoked per day were not different between the groups.

### 3.1. Between-Group Comparisons of Regional mGluR5 Availability

The ROI-based analysis using the two-tailed t-test showed no significant group differences in regional mGluR5 availability in any individual ROI, although we do note that, with the single exception of globus pallidus, the mean BP_ND_ was numerically lower in the alcohol group that in the control group (Table 2).

**Figure 2:**
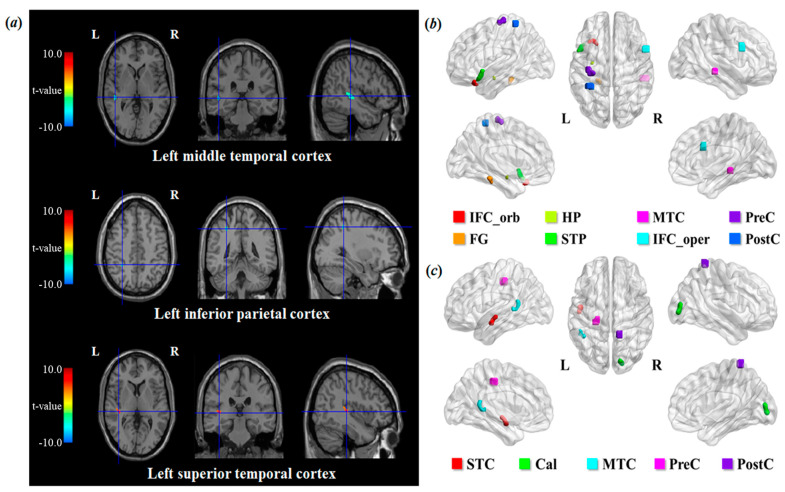
Results of the voxel-based between-group analysis (**a**) and correlation analysis between clinical variables and regional mGluR5 availability (**b**,**c**). The [^11^C]ABP688 BP_ND_ of the alcohol dependence group was significantly lower in the left middle temporal and inferior parietal cortices and significantly higher in the left superior temporal cortex (uncorrected *p* < 0.001) (**a**). The voxel-based analysis with age and the number of cigarettes smoked per day as covariates showed that [^11^C]ABP688 BP_ND_ had significant positive correlations with the duration of alcohol abstinence as shown in (**b**) (uncorrected *p* < 0.001) and negative correlations with the duration of illness as shown in (**c**) (uncorrected *p* < 0.001). BP_ND_, binding potential with respect to nondisplaceable compartment; Cal, calcarine fissure and surrounding cortex; FG, fusiform gyrus; HP, hippocampus; IFC, inferior frontal cortex; MTC, middle temporal cortex; Oper, opercular part; Orb, orbital part; STC, superior temporal cortex; STP, superior temporal pole; PreC, precentral cortex; PostC, postcentral cortex.

The voxel-based analysis showed that [^11^C]ABP688 BP_ND_ values were significantly lower in the alcohol dependence group than in the control group in the left middle temporal cortex and left inferior parietal cortex (*p* < 0.001) (Table 3; Figure 2a). In addition, the [^11^C]ABP688 BP_ND_ value in the left superior temporal cortex was significantly higher in the alcohol dependence group than in the control group (*p* < 0.001) (Table 3; Figure 2a).

### 3.2. Correlation between Clinical Variables and Regional mGluR5 Availability

Age and the number of cigarettes smoked per day were both significantly negatively correlated with [^11^C]ABP688 BP_ND_ values in widespread regions in the voxel-based analysis for whole subjects (*n* = 22) at *p* < 0.005 (Supplementary Table S1; Supplementary Figure S2). The ROI-based analysis also showed significant negative correlations for the entire subject group (*n* = 22) between [^11^C]ABP688 BP_ND_ and the number of cigarettes smoked per day in widespread regions, including the anterior cingulate gyrus, frontal cortex, parietal cortex, temporal cortex, amygdala, thalamus, caudate, putamen, and ventral striatum, at *p* < 0.05 (Supplementary Table S2). Age was not significantly correlated with [^11^C]ABP688 BP_ND_ in the ROI-based analysis (Supplementary Table S2).

In the initial ROI-based analysis using Pearson’s correlation coefficients, the duration of alcohol abstinence was significantly positively correlated with [^11^C]ABP688 BP_ND_ values in the anterior cingulate gyrus (*r* = 0.61, *p* = 0.04), inferior parietal cortex (*r* = 0.64, *p* = 0.03), and amygdala (*r* = 0.58, *p* < 0.05). However, upon exclusion of the two subjects with very long-term abstinence (113 and 120 months abstinence) from the analysis, these correlations were no longer significant (*p*-values > 0.05). The correlations for duration of abstinence also lost statistical significance after controlling for the number of cigarettes smoked per day using partial correlation analysis (Supplementary Table S3). No other significant correlations were observed in the ROI-based correlation analysis (Supplementary Table S3).

The voxel-based analysis with age and the number of cigarettes smoked per day as covariates revealed that the duration of alcohol abstinence was significantly positively correlated with [^11^C]ABP688 BP_ND_ values in regions, including the inferior frontal cortex, middle temporal cortex, temporal pole, precentral and postcentral cortices, and hippocampus (*p* < 0.001) (Table 4; Figure 2b). In accord with the ROI-based analysis, these voxelwise correlations of [^11^C]ABP688 BP_ND_ with the duration of alcohol abstinence were no longer significant when the two subjects with very long term abstinence were excluded from the analysis (*p*-values > 0.005). The voxel-based analysis using age and smoking as covariates showed significant negative correlations between duration of illness and [^11^C]ABP688 BP_ND_ values in the middle temporal cortex, superior temporal cortex, and precentral and postcentral cortices (*p* < 0.001) (Table 4; Figure 2c). The OCDS and MAST scores did not show any significant correlations with [^11^C]ABP688 BP_ND_, although they had some negative and positive correlations with [^11^C]ABP688 BP_ND_ in several regions (*p* < 0.005) (Table 4).

### 3.3. [^11^C]ABP688 BP_ND_ Seed-Based rs-Functional Connectivity

**Figure 3:**
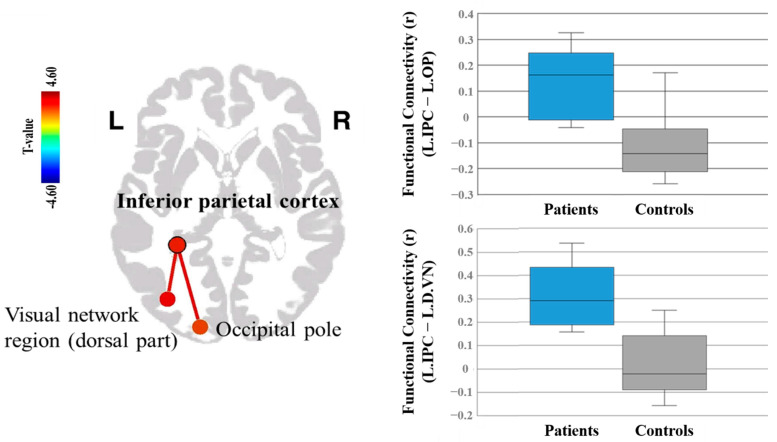
[^11^C]ABP688 BP_ND_ seed-based resting-state functional connectivity. The LIPC to the LOP showed positive connectivity (positive correlation) in the alcohol dependence group, while it showed negative connectivity (anticorrelation) in the control group (*p* = 0.0002, FDR*p* = 0.027). The LIPC to the LDVN showed significant positive connectivity (positive correlation) in the alcohol dependence group compared to the control group (*p* = 0.0003, FDR*p* = 0.027); LIPC, left inferior parietal cortex; LOP, left occipital pole; LDVN, left visual network region (dorsal part), FDR*p*, false discovery rate—corrected *p*-value.

As shown in Table 3, the clusters derived from the voxel-based between-group comparisons of [^11^C]ABP688 BP_ND_ were located in the left inferior parietal cortex, left superior temporal cortex, and left middle temporal cortex. These clusters were defined as the seed regions for rs-functional connectivity analysis. The two-sample t-test revealed significant group differences in the left inferior parietal cortex seed. The left inferior parietal cortex to the left occipital pole showed positive connectivity (positive correlation) in the alcohol dependence group, while it showed negative connectivity (anticorrelation) in the control group (*p* = 0.0002, false discovery rate—corrected *p*-value (FDR*p*) = 0.027). The left inferior parietal cortex to the dorsal visual cortex (network region) showed significant positive connectivity (positive correlation) in the alcohol dependence group compared to the control group (*p* = 0.0003, FDR*p* = 0.027) (Table 5; Figure 3).

## 4. Discussion

In the present study, we quantitatively analyzed mGluR5 availability in prolonged abstinent males with alcohol dependence using [^11^C]ABP688 PET to search for alterations of mGluR5 availability. In addition, we conducted rs-fMRI in the same subjects and performed resting-state functional connectivity analysis to investigate the functional significance of the alterations of mGluR5 availability. In the voxel-based analysis, we found region-specific alterations in mGluR5 availability in the prolonged abstinent subjects. Furthermore, [^11^C]ABP688 BP_ND_ seed-based rs-fMRI analysis revealed significant alterations of functional connectivity in these subjects. Overall, these results suggest the possibility of altered in vivo mGluR5 availability and related functional connectivity changes in our group of abstinent male subjects. To our knowledge, this is the first report on the combined analysis of in vivo mGluR5 PET imaging and seed-based fMRI in subjects with alcohol dependence, thus providing new information on integrated molecular and systems-level changes in prolonged abstinent subjects with alcohol dependence. However, due to the small sample size, the results of our study should be considered as preliminary.

The voxel-based analysis revealed significantly lower [^11^C]ABP688 BP_ND_ values in the alcohol dependence group in the left middle temporal and inferior parietal cortices, although there were no significant group differences in the ROI-based analysis, despite low-to-medium effect sizes, which suggested globally lower [^11^C]ABP688 BP_ND_ in the alcohol dependence group. On the other hand, the voxel-based analysis also indicated significantly higher [^11^C]ABP688 BP_ND_ in the left superior temporal cortex in the alcohol dependence group. These results suggest the possibility of region-specific alterations of mGluR5 availability in our group of abstinent subjects, which is in line with findings of preclinical studies showing region-specific neuroadaptive alterations of glutamatergic protein expression related to ethanol exposure [60,61]. Given rodent findings that mGluR5 antagonists attenuate cue-conditioned alcohol-seeking behaviors, the present observations of altered mGluR5 availability in some cortical regions may indicate neuroadaptive changes of the mGluR5-protein kinase C (PKC) signaling pathway [62]. The involvement of temporo-parietal cortex suggests the possibility of altered cortical function involving multiple processes, such as information processing, cognitive control, and response inhibition [63], which might be associated with adaptive functional changes in subjects with prolonged abstinence.

The number of cigarettes smoked per day was significantly negatively correlated with [^11^C]ABP688 BP_ND_ values in widespread regions. These results are in line with previous reports of [^11^C]ABP688 PET studies, which showed decreased mGluR5 binding associated with cigarette smoking in humans [19,20,64]. Since all smokers in the alcohol group had been chronic smokers for more than five years (mean duration of smoking: 22.2 (SD = 8.1) years), our results may reflect a downregulation process associated with chronic nicotine use, as previously suggested [19,20]. Since only three subjects of the alcohol group were nonsmokers, we could not examine the effects of smoking on the relationship between alcohol dependence and mGluR5 availability. Further studies are necessary to examine whether there is a significant interaction effect between nicotine use and alcohol dependence on mGluR5 availability.

The correlations with clinical characteristics should be interpreted with caution, given the small sample size and the preliminary nature of the analysis without corrections for multiple correlations. However, we note that the voxel-based analysis showed significant positive correlations between mGluR5 availability and duration of alcohol abstinence. Akkus et al. [64] reported that long-term ex-smokers and individuals who had never smoked showed no significant differences in mGluR5 availability, suggesting recovery of smoking-related mGluR5 changes. This mirrors findings in a few longitudinal PET studies on alcohol dependence. In the [^18^F]FPEB PET study of recently detoxified alcohol-dependent patients, in which mGluR5 availability was measured at the two- and six-month follow-up visits, there was sustained recovery of mGluR5 availability in corticolimbic regions [24]. Similarly, a prospective [^18^F]fallypride PET study showed significant recovery of striatal dopamine D_2/3_ receptor availability in a subgroup with one-year abstinence [65]. Further prospective studies are required to clearly determine the time course of mGluR5 recovery in different brain regions as a function of alcohol abstinence period, and whether the restoration can serve as a significant predictor of clinical recovery in alcohol dependence.

Our seed-based functional connectivity analysis using rs-fMRI data with clusters derived from between-group comparisons of [^11^C]ABP688 BP_ND_ as seeds indicated possible perturbations in the functional connectivity between parietal and occipital cortices (putative vision-associated areas). These results are in line with previous findings of significantly higher resting-state functional connectivity in abstinent alcohol-dependent subjects [26,66,67]. In particular, the cortical regions in our study showing significantly altered functional connectivity with the seed in the alcohol dependence group were primarily nodes of visual attention networks. This result may provide insight into the neurobiological mechanisms associated with alterations in segregating relevant stimuli and switching from interoceptive desires and cravings to cognitive control in abstinent subjects [68]. The present results, along with previous reports, indicate altered neural networks in abstinent subjects with alcohol dependence. A much larger group size with multimodal imaging data including diffusion tensor imaging in addition to molecular imaging would be required to confirm the associations between mGluR5 availability and structural networks in alcoholic individuals. Also, critically sampled fast MRI data would enable stronger statistical inferences on functional connectivity in the absence of physiological signal aliasing [69].

The inferior parietal cortex seed region is among the integral regions comprising the default mode network (DMN) [70,71]. Since glutamate transmission may contribute to the neural basis of DMN [72], our results imply that mGluR5 transmission may contribute to the altered DMN connectivity observed in the abstinent subjects [73], perhaps in relation to compensatory mechanisms. Since our subjects had maintained prolonged abstinence, our results may also suggest the possible development of neuroadaptive changes, which could relate to the reorganization of functional networks [26]. Our findings are also in line with the notion that adaptive changes in the glutamatergic system play an important role in alcohol use disorders [74]. Understanding neuroadaptive patterns during dependence and abstinence may provide insights into alcohol-related behavioral changes, and the potential impact of these neuroadaptive patterns on long-term abstinence and recovery from alcohol addiction.

In our study, only male subjects were enrolled and scanned. Preclinical studies using the intermittent ethanol exposure paradigm showed an upregulation of *N*-methyl-*D*-aspartate (NMDA) receptors in the extended amygdala region in adolescent male mice without any such changes in female mice [75]. Furthermore, binge ethanol drinking reduced mGluR5 gene expression in the nucleus accumbens in male but not female mice [76]. Based on these preclinical findings, we speculate that mGluR5 availability would differ in limbic regions between male and female subjects with alcohol dependence.

Our main PET finding was altered mGluR5 availability in voxel clusters of the parietal and temporal cortex in the alcohol dependence group. Preclinical findings have implicated glutamatergic alterations in the PFC and limbic areas in alcohol dependence, and the PFC has strong glutamatergic connections with parietal and temporal cortex [77]. We note that preclinical addiction models may not fully emulate human conditions or pathophysiology [5]. Future studies with glutamate and dopamine PET imaging might illuminate the downstream effects of altered cortical mGluR5 availability on mesolimbic dopaminergic signaling.

The interpretation of the present results should be considered in the light of some limitations. The relatively small sample size was underpowered to detect significant group differences in the ROI-based analysis. The region with the largest effect size in the ROI-based analysis was the hippocampus (Cohen’s *d* = 0.66), where the mean BP_ND_ was 16% lower in the alcohol dependence group than in the control group. Power analysis predicts that a sample size of at least 32 (alcohol group) and 26 (control group) subjects would be required to detect a significant difference of this magnitude (power = 0.8, alpha = 0.05). Since our subjects were all males with prolonged abstinence, the findings may not be generalizable to a more heterogeneous group of subjects with alcohol dependence. Given the numerous functions that are attributed to glutamate signaling at mGluR5, the present exploratory investigation of mGluR5 availability in a relatively small population of subjects with prolonged alcohol abstinence should eventually be followed by studies using larger numbers of subjects and controls. Other factors, such as age, sex, smoking habits, social and family status, body mass index, and lifestyle, in addition to the duration of abstinence, should be considered in future studies. The [^11^C]ABP688 BP_ND_ was derived using a reference tissue model, rather than the gold standard metabolite-corrected arterial input-based quantification using a two-tissue compartment (2TC) model [39,40]. However, arterial blood sampling is invasive and is associated with discomfort and potential adverse effects, which would have further limited recruitment of subjects. We used the cerebellar gray matter reference region as previously suggested for this tracer [39,40,41]. Despite the lack of any brain region completely devoid of mGluR5, postmortem studies have reported negligible specific binding in the cerebellar gray matter [44,45,46]. Although cerebellar gray matter has been validated as an appropriate reference region for [^11^C]ABP688 PET by displacement studies in the brains of rodents and nonhuman primates [42,43], these and the present results should be interpreted with caution. Quantitative binding studies in vitro indicate that specific mGluR5 binding of [^18^F]F-PEB in cerebellum gray matter relative to that in forebrain differs across species, ranging from 5% in rodent to as much as 15% in human postmortem samples [78]. Others report a displaceable [^11^C]ABP688 binding component in nonhuman primate cerebellum of about 10% that seen in forebrain [40]. It follows that our method may slightly underestimate BP_ND_.

## 5. Conclusions

Using a multimodal imaging approach combining [^11^C]ABP688 PET and rs-fMRI, our study provides new information on the integrated molecular and systems-level changes associated with alcohol dependence. The results of the present preliminary study suggest an involvement of mGluR5 in alcohol dependence, and hint at an association between mGluR5 changes and functional connectivity. We see grounds for undertaking a larger-scale longitudinal study to investigate the effect of duration of abstinence on these findings, as in an earlier PET study of dopamine D_2/3_ receptors [65].

## Figures and Tables

**Table 1:** Demographic/clinical characteristics and PET scan parameters.

Variables	Alcohol Group (N = 12)	Control Group (N = 10)	*p*-Value
Age (years) ^a^	45.7 ± 7.2	39.8 ± 7.9	0.084
Smoker/Nonsmoker	9/3	5/5	0.225
Number of cigarettes smoked (/day) ^a,b^	10.8 ± 9.5	5.1 ± 7.4	0.139
Duration of illness (years)^a^	7.2 ± 3.9	-	-
Duration of alcohol abstinence (months) ^a^	28.4 ± 43.0	-	-
OCDS ^a^	11.2 ± 7.8	-	-
MAST ^a^	40.6 ± 5.7	-	-
Injected Dose (MBq) ^a^	607 ± 58	595 ± 100	0.731
Specific Activity (GBq/umol) ^a^	20.2 ± 9.1	12.9 ± 7.9	0.083

^a^ Numbers are presented as mean ± standard deviation (SD). ^b^ Number of cigarettes smoked (/day) per member of each group. OCDS, Obsessive Compulsive Drinking Scale; MAST, Michigan Alcoholism Screening Test.

**Table 2:** ROI-based between-group comparisons of regional [^11^C]ABP688 BP_ND_ values.

ROIs	[^11^C]ABP688 BP_ND_ Value		*p*-Value	Effect Size (Cohen’s *d*)
	Alcohol Group Mean (SD)	Control Group Mean (SD)		
ACG	0.521 (0.171)	0.598 (0.182)	0.321	0.435
SFC	0.447 (0.141)	0.470 (0.133)	0.705	0.164
MFC	0.487 (0.152)	0.520 (0.143)	0.615	0.219
IFC	0.467 (0.144)	0.527 (0.139)	0.331	0.427
STC	0.525 (0.142)	0.606 (0.140)	0.195	0.573
MTC	0.518 (0.156)	0.575 (0.142)	0.383	0.382
SPC	0.300 (0.077)	0.342 (0.098)	0.275	0.480
IPC	0.492 (0.140)	0.543 (0.137)	0.394	0.373
Hip	0.459 (0.116)	0.544 (0.141)	0.137	0.662
Amy	0.577 (0.174)	0.660 (0.164)	0.271	0.485
Tha	0.325 (0.083)	0.359 (0.099)	0.394	0.372
Cau	0.465 (0.143)	0.531 (0.162)	0.319	0.437
Put	0.675 (0.181)	0.725 (0.161)	0.506	0.290
VS	0.492 (0.154)	0.576 (0.163)	0.230	0.530
GP	0.352 (0.105)	0.331 (0.068)	0.587	0.237

ROI, region of interest; BP_ND_, binding potential with respect to nondisplaceable compartment; SD, standard deviation; ACG, anterior cingulate gyrus; SFC, superior frontal cortex; MFC, middle frontal cortex; IFC, inferior frontal cortex; STC, superior temporal cortex; MTC, middle temporal cortex; SPC, superior parietal cortex; IPC, inferior parietal cortex; Hip, hippocampus; Amy, amygdala; Tha, thalamus; Cau, caudate; Put, putamen; VS, ventral striatum; GP, globus pallidus.

**Table 3:** Voxel-based between-group comparisons of [^11^C]ABP688 BP_ND_ values.

MNI Coordinate	Regions	Cluster Size	Cluster Volume (mm^3^)	[^11^C]ABP688 BP_ND_ (Mean ± SD)		T-Value	*p*-Value (Uncorr.)	FDR*p*
				Alcohol Group	Control Group			
Alcohol < Control								
−48; −30; 2	Left MTC	124	419	0.316 ± 0.171	0.633 ± 0.176	4.602	0.0001	0.463
−32; −44; 47	Left IPC	25	84	0.341 ± 0.125	0.584 ± 0.169	3.957	0.0004	0.463
Alcohol > Control								
−42; −27; 5	Left STC	80	270	0.414 ± 0.074	0.257 ± 0.064	5.951	<0.0001	0.447

BP_ND_, binding potential with respect to nondisplaceable compartment; SD, standard deviation; MNI, Montreal Neurological Institute; FDR*p*, false discovery rate—corrected *p*-value; MTC, middle temporal cortex; IPC, inferior parietal cortex; STC, superior temporal cortex.

**Table 4:** Results of voxel-based correlation analysis between [^11^C]ABP688 BP_ND_ values and clinical variables in the alcohol dependence group.

Clinical Variables	MNI Coordinate	Regions	Cluster Size	Cluster Volume (mm^3^)	T-Value	*p*-Value (Uncorr.)	FDR*p*
Duration of illness							
Negative correlation	14; −45; 72	Right postcentral cortex	32	108	5.515	<0.001	0.176
	17; −87; 2	Right calcarine fissure and surrounding cortex	91	307	8.424		0.176
	−44; −48; 14	Left middle temporal cortex	131	442	8.305		0.176
	−44; −6; 60	Left precentral cortex	26	88	7.167		0.176
	−48; −6; −15	Left middle temporal cortex and superior temporal cortex	44	149	7.456		0.176
Duration of alcohol abstinence							
Positive correlation	53; −30; −6	Right middle temporal cortex	30	101	5.879	<0.001	0.365
	56; 14; 32	Right inferior frontal cortex (opercular part)	34	115	6.789		0.361
	−35; −42; 65	Left postcentral cortex	24	81	7.058		0.361
	−38; −17; 68	Left precentral cortex	53	179	6.969		0.361
	−50; 14; −12	Left superior temporal pole	117	395	10.365		0.361
	−32; −8; −17	Left hippocampus	32	108	9.016		0.361
	−20; −36; −18	Left fusiform gyrus	35	118	5.458		0.377
	−27; 24; −24	Left inferior frontal cortex (orbital part) and superior temporal pole	142	479	8.691		0.361
OCDS ^a^							
Negative correlation	−21; 48; 30	Left middle frontal cortex	32	108	4.668	<0.005	0.514
	−62; −11; 39	Left postcentral cortex	28	95	9.347		0.514
	−39; −29; 6	Left Rolandic operculum, superior temporal cortex, and Heschl’s gyrus	125	422	5.498		0.514
MAST ^a^							
Positive correlation	38; 23; 21	Right inferior frontal cortex (triangular part)	36	122	4.029	<0.005	0.554
	−32; −66; 32	Left middle occipital cortex	113	381	5.324		0.554

These voxel-based analyses were performed using age and the number of cigarettes smoked per day as covariates. ^a^ No significant correlations were found at *p* < 0.001. BP_ND_, binding potential with respect to nondisplaceable compartment; FDR*p*, false discovery rate—corrected *p*−value; MNI, Montreal Neurological Institute; OCDS, Obsessive Compulsive Drinking Scale; MAST, Michigan Alcoholism Screening Test.

**Table 5:** Between-group comparisons of [^11^C]ABP688 BP_ND_ seed-based resting-state functional connectivity.

Seed	Regions	Functional Connectivity		*p*−Value (Uncorr.)	FDR*p*
		Alcohol Mean (SD)	Control Mean (SD)		
Left middle temporal cortex	Left insular	0.10 (0.16)	−0.06 (0.23)	0.002	0.229
	Right temporal pole	0.28 (0.10)	0.12 (0.14)	0.008	0.358
	Left salience network (anterior insular part)	0.34 (0.15)	0.15 (0.16)	0.010	0.358
Left inferior parietal cortex	Left occipital pole	0.14 (0.13)	−0.11 (0.13)	<0.001	0.027
	Visual network (dorsal part)	0.34 (0.19)	0.02 (0.15)	<0.001	0.027
	Left lateral inferior occipital cortex	0.28 (0.17)	0.05 (0.19)	0.006	0.334
Left superior temporal cortex	Visual network (ventral part)	0.14 (0.12)	−0.10 (0.19)	0.002	0.280
	Left occipital pole	0.13 (0.13)	−0.11 (0.21)	0.005	0.280
	Right occipital fusiform gyrus	0.04 (0.09)	−0.10 (0.12)	0.005	0.280

BP_ND_, binding potential with respect to nondisplaceable compartment; SD, standard deviation; FDR*p*, false discovery rate—corrected *p*−value.

## Data Availability

The data presented in this study are available upon reasonable request from the corresponding authors. The data are not publicly available due to the containing information that could compromise the privacy of research participants.

## Supplementary Materials

The following are available online at https://www.mdpi.com/article/10.3390/life11060506/s1, Table S1: Voxel-based correlation analysis between [^11^C]ABP688 BP_ND_ values and age/number of cigarettes smoked per day in whole subjects, Table S2: ROI-based correlation analyses between [^11^C]ABP688 BP_ND_ values and age/number of cigarettes smoked per day in whole subjects, Table S3: ROI-based correlation analysis between [^11^C]ABP688 BP_ND_ values and clinical characteristics in the alcohol dependence group, Figure S1: Comparisons of the cerebellar standard uptake value (SUV) and cerebellar gray matter (GM) volume between the alcohol dependence and control groups, Figure S2: Voxel-based analysis showing negative correlations of [^11^C]ABP688 BP_ND_ with age and the number of cigarettes smoked per day in whole subjects.
