# Supplementary material for: Functional Analysis of Brain Imaging Suggests Changes in the Availability of mGluR5 and Altered Connectivity in the Cerebral Cortex of Long-Term Abstaining Males with Alcohol Dependence: A Preliminary Study
Source: Life (Basel). 2021 May 30;11(6):506. doi: 10.3390/life11060506 (PMC8228527; doi:10.3390/life11060506)
Supplement: Supplementary file 1 [file life-11-00506-s001.zip › life-1185103-supplementary.pdf]

Supplementary Material

Functional Analysis of Brain Imaging Suggests Changes in the Availability of mGluR5 and Altered Connectivity in the Cerebral Cortex of Long-Term Abstaining Males with Alcohol Dependence: A Preliminary Study

Table S1. Voxel-based correlation analysis between [^{11}C]ABP688 BP_{ND} values and age/number of cigarettes smoked per day in whole subjects (n = 22).

Variables	MNI Coordinates	Regions	T–Value	*p*–Value	FDR*p*
Number of cigarettes smoked per day					
Negative correlation	32;−81;−23	Right inferior temporal cortex, middle temporal cortex, inferior occipital cortex, and fusiform gyrus	5.773	<0.0005	0.030
	18;11;−21	Right parahippocampal gyrus and olfactory cortex	4.583		0.030
	42;0;−47	Right inferior temporal cortex	5.238		0.030
	5;8;59	Right supplementary motor area and superior frontal cortex (medial part)	5.253		0.030
	17;21;63	Right supplementary motor area	4.676		0.030
	56;−47;48	Right inferior parietal cortex	4.461		0.030
	41;14;35	Right inferior frontal cortex (opercular part)	4.486		0.030
	62;9;27	Right precentral gyrus and postcentral gyrus	4.296		0.030
	57;2;−5	Right superior temporal cortex and superior temporal pole	4.578		0.030
	53;41;11	Right inferior frontal cortex (triangular part)	4.888		0.030
	59;9;−23	Right middle temporal pole	4.130		0.030
	62;−5;−26	Right middle temporal cortex	4.567		0.030
	47;29;−12	Right inferior frontal cortex (orbital part)	4.230		0.030
	12;−98;8	Right cuneus	4.417		0.030
	3;−12;33	Right middle cingulate gyrus	4.205		0.030
	2;−57;38	Left precuneus and right precuneus	4.690		0.030
	0;56;11	Left superior frontal cortex (medial part) and right superior frontal cortex (medial part)	4.216		0.030
	3;45;45	Left superior frontal cortex (medial part) and right superior frontal cortex (medial part)	5.073		0.030
	0;2;38	Right middle cingulate gyrus and left middle cingulate gyrus	5.644		0.030
	−3;66;17	Left superior frontal cortex (medial part)	4.684		0.030
	2;38;17	Left anterior cingulate cortex	4.345		0.030
	−12;−65;39	Left precuneus and cuneus	5.343		0.030
	−5;35;50	Left superior frontal cortex (medial part)	5.001		0.030
	−2;−27;44	Left middle cingulate cortex	4.547		0.030

	−14;8;14	Left caudate	4.657		0.030
	−17;5;−29	Left parahippocampal gyrus	4.390		0.030
	−20;−42;−14	Left fusiform gyrus	4.286		0.030
	−38;−83;−6	Left inferior occipital cortex	4.343		0.030
	−30;−90;3	Left middle occipital cortex	4.342		0.030
	−6;9;72	Left supplementary motor area	5.203		0.030
	6;−92;−6	Left calcarine fissure and surrounding cortex	4.175		0.030
	−3;62;−6	Left medial orbitofrontal cortex	4.368		0.030
Age					
Negative correlation	59;−11;47	Right precentral gyrus	4.025		0.503
	27;18;65	Right superior frontal cortex	3.746		0.503
	35;−39;−8	Right parahippocampal gyrus	4.411		0.503
	44;−33;11	Right superior temporal cortex	3.367		0.503
	−2;−84;32	Left cuneus	3.496		0.503
	−2;46;36	Left superior frontal cortex (medial part)	3.120		0.503
	−2;−59;11	Left precuneus and calcarine fissure and surrounding cortex	4.016		0.503
	−36;20;29	Left inferior frontal cortex (triangular part)	3.821	<0.005	0.503
	−53;−24;26	Left supramarginal gyrus and postcentral gyrus	3.533		0.503
	−24;15;−30	Left superior temporal pole	4.137		0.503
	−45;21;−18	Left superior temporal pole	3.305		0.503
	3;14;−2	Left olfactory cortex	3.342		0.503
	−48;15;−11	Left superior temporal pole	3.275		0.503
	−14;−32;6	Left thalamus	3.749		0.503
	−27;−14;−15	Left hippocampus	4.156		0.503

BP_{ND}, binding potential with respect to nondisplaceable compartment; MNI, Montreal Neurological Institutel; FDR*p*, false discovery rate—corrected *p*–value.

Table S2. ROI-based correlation analyses between [11C]ABP688 BP_{ND} values and age/number of cigarettes smoked per day in whole subjects (n = 22).

Regions of Interest	Number of Cigarettes Smoked Per Day		Age	
	**Pearson Correlation**	***p*–Value**	**Pearson Correlation**	***p*–Value**
Anterior cingulate gyrus	−0.567	0.006*	−0.221	0.322
Superior frontal cortex	−0.416	0.054	−0.172	0.443
Middle frontal cortex	−0.472	0.027*	−0.154	0.495
Inferior frontal cortex (triangular part)	−0.465	0.029*	−0.207	0.356
Superior temporal cortex	−0.537	0.010*	−0.253	0.256
Middle temporal cortex	−0.535	0.010*	−0.164	0.466
Superior parietal cortex	−0.472	0.026*	−0.307	0.164
Inferior parietal cortex	−0.536	0.010*	−0.194	0.387
Hippocampus	−0.420	0.052	−0.383	0.078
Amygdala	−0.459	0.032*	−0.237	0.289
Thalamus	−0.460	0.031*	−0.306	0.166
Caudate	−0.539	0.010*	−0.297	0.179
Putamen	−0.477	0.025*	−0.117	0.605
Ventral striatum	−0.572	0.005*	−0.308	0.163
Globus pallidus	−0.251	0.259	0.138	0.541

*The asterisk indicates a significant correlation ($p < 0.05$). ROI, region of interest; BP_{ND}, binding potential with respect to nondisplaceable compartment.

Table S3. ROI-based correlation analysis between [^{11}C]ABP688 BP_{ND} values and clinical characteristics in the alcohol dependence group (*n* = 12).

ROIs	Duration of Illness			Duration of Alcohol Abstinence			OCDS			MAST		
	r	*p*	*p'*	*r*	*p*	*p'*	*r*	*p*	*p'*	*r*	*p*	*p'*
ACG	−0.177	0.582	0.240	0.606	0.037*	0.164	−0.113	0.727	0.312	0.055	0.865	0.815
SFC	−0.402	0.195	0.094	0.466	0.127	0.268	−0.178	0.581	0.369	0.133	0.681	0.664
MFC	−0.341	0.278	0.121	0.513	0.088	0.233	−0.176	0.584	0.322	0.102	0.753	0.725
IFC	−0.350	0.265	0.150	0.471	0.122	0.246	−0.227	0.477	0.295	0.108	0.738	0.724
STC	−0.243	0.447	0.232	0.498	0.100	0.270	−0.152	0.636	0.353	0.081	0.803	0.773
MTC	−0.220	0.492	0.247	0.557	0.060	0.189	−0.171	0.595	0.295	0.004	0.990	0.965
SPC	−0.270	0.396	0.183	0.565	0.056	0.173	−0.095	0.770	0.453	0.042	0.897	0.868
IPC	−0.212	0.507	0.233	0.637	0.026*	0.103	−0.151	0.639	0.299	0.013	0.968	0.938
Hip	−0.248	0.437	0.301	0.534	0.073	0.142	−0.170	0.597	0.415	−0.038	0.907	0.923
Amy	−0.275	0.387	0.241	0.578	0.049*	0.109	−0.199	0.535	0.338	−0.168	0.601	0.617
Tha	−0.057	0.860	0.597	0.514	0.088	0.238	−0.287	0.366	0.154	0.143	0.657	0.625
Cau	−0.217	0.499	0.224	0.532	0.075	0.254	−0.131	0.686	0.335	−0.012	0.970	0.995
Put	−0.329	0.297	0.162	0.560	0.058	0.136	−0.200	0.534	0.324	0.010	0.975	0.957
VS	−0.217	0.497	0.225	0.496	0.101	0.324	−0.131	0.685	0.336	0.174	0.588	0.534
GP	−0.336	0.286	0.197	0.511	0.089	0.148	−0.282	0.375	0.253	0.116	0.719	0.714

*The asterisk indicates a significant correlation ($p < 0.05$). The *p'* indicates the *p*–value of partial correlation analysis after controlling for the number of cigarettes smoked per day. ROI, region of interest; BP_{ND}, binding potential with respect to nondisplaceable compartment; OCDS, Obsessive Compulsive Drinking Scale; MAST, Michigan Alcoholism Screening Test; ACG, anterior cingulate gyrus; SFC, superior frontal cortex; MFC, middle frontal cortex; IFCt, inferior frontal cortex (triangular part); STC, superior temporal cortex; MTC, middle temporal cortex; SPC, superior parietal cortex; IPC, inferior parietal cortex; Hip, hippocampus; Amy, amygdala; Tha, thalamus; Cau, caudate; Put, putamen; VS, ventral striatum; GP, globus pallidus.

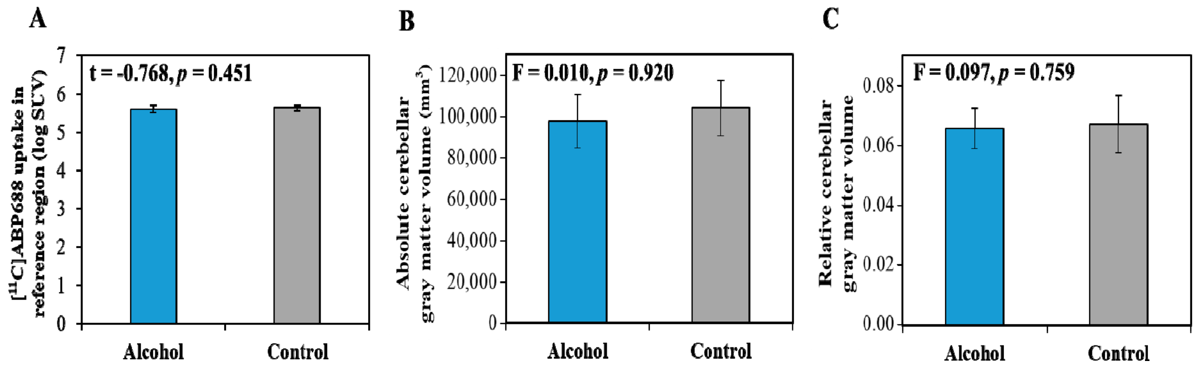

Figure S1. Comparisons of the cerebellar standard uptake value (SUV) and cerebellar gray matter (GM) volume between the alcohol dependence and control groups. The [^{11}C]ABP688 SUV in the cerebellar GM (reference region) did not differ significantly between the groups (log SUV: alcohol group: 5.598 ± 0.087, control group: 5.624 ± 0.069; $t = -0.768$, $p = 0.451$) (A). The cerebellar GM volume was compared between the groups using analysis of covariance (ANCOVA) with age as a covariate. There was no significant between-group difference in absolute cerebellar GM volume (alcohol group: 97513.8 ± 12996.6 mm^3, control group: 103881.2 ± 13631.8 mm^3, $F = 0.010$, $p = 0.920$) (B). No significant group difference was observed in relative cerebellar GM volume, which was calculated by dividing the absolute cerebellar GM volume by the intracranial volume (alcohol group: 0.066 ± 0.007, control group: 0.067 ± 0.009, $F = 0.097$, $p = 0.759$) (C).

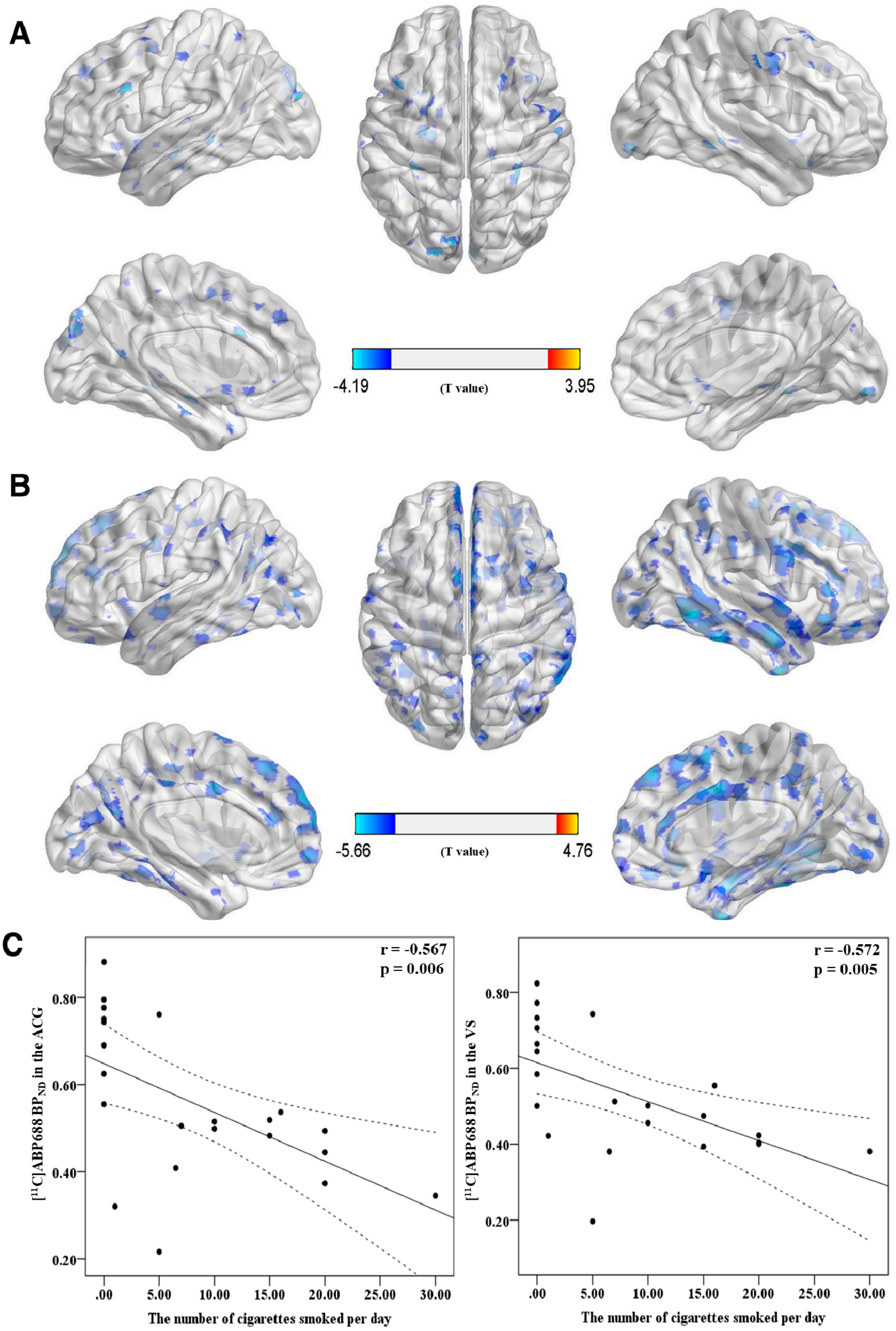

Figure S2. Voxel-based analysis showing negative correlations of [^{11}C]ABP688 BP_{ND} with age (p < 0.005, k = 20) (A) and the number of cigarettes smoked per day (p < 0.0005, k = 20) (B) in whole subjects (n = 22). Images were visualized with the BrainNet Viewer (http://www.nitrc.org/projects/bnv/). Representative scatter plots of the results of region-of-interest (ROI)-based correlation analysis between [^{11}C]ABP688 BP_{ND} and the number of cigarettes smoked per day for the anterior cingulate gyrus (ACG) and ventral striatum (VS) (p < 0.01) (C).
